# Psychophysical Differences in Ventilatory Awareness and Breathlessness between Athletes and Sedentary Individuals

**DOI:** 10.3389/fphys.2016.00231

**Published:** 2016-06-16

**Authors:** Olivia K. Faull, Pete J. Cox, Kyle T. S. Pattinson

**Affiliations:** ^1^FMRIB Centre and Nuffield Division of Anesthetics, Nuffield Department of Clinical Neurosciences, University of OxfordOxford, UK; ^2^Department of Physiology, Anatomy and Genetics, University of OxfordOxford, UK

**Keywords:** breathlessness, anxiety, ventilation, athletes, maximal exercise

## Abstract

**Purpose:** Breathlessness is a complex set of symptoms that are comprised of both sensory and affective (emotional) dimensions. While ventilation is now understood to be a potential limiter to performance in highly-trained individuals, the contribution of breathlessness-anxiety in those nearing maximal ventilation during intense exercise has not yet been considered as a limiter to performance.

**Methods:** In this study, we compared the physiology and psychology of breathlessness in 20 endurance athletes with 20 untrained age- and sex-matched sedentary controls. Subjects completed baseline spirometry and anxiety questionnaires, an incremental exercise test to exhaustion and a steady-state hypercapnic ventilatory response test, with concurrent measures of breathlessness intensity and breathlessness-anxiety.

**Results:** Compared with sedentary subjects, athletes reported equivalent breathlessness intensity but greater breathlessness-anxiety at maximal exercise (athletes vs. sedentary (mean ± SD): breathlessness intensity (0–100%) 80.7 (22.7) vs. 72.5 (17.2), *p* = 0.21; breathlessness-anxiety (0–100%), 45.3 (36.3) vs. 22.3 (20.0), *p* = 0.02). Athletes operated at higher proportions of their maximal ventilatory capacity (MVV) (athletes vs. sedentary (mean ventilation ± SD; % MVV): 101.6 (27.2) vs. 73.7 (30.1), *p* = 0.003). In the athletes there was a positive linear correlation between ventilation and breathlessness score during the hypercapnic challenge that was not observed in the sedentary controls.

**Conclusion:** The results of this study indicate that whilst operating at high proportions of maximal ventilation, breathlessness-anxiety becomes increasingly prominent in athletes. Our results suggest that ventilatory perception pathways may be a target for improved athletic performance in some individuals.

## Introduction

Breathlessness is the frightening sensation of not getting enough air. A recent model of breathlessness describes multiple sensory dimensions that include work/effort, air hunger and chest tightness (Schwartzstein et al., 1990; Lansing et al., 2000, 2009). As discussed by Lansing et al. (2009), breathlessness has both sensory components (often related to the strength or intensity of the sensation) and affective or emotional components that relate to how unpleasant or worrying the sensation feels, and to the resulting associated anxiety (termed breathlessness-anxiety in this paper). These sensory and affective components may operate independently in the face of changes in ventilation (von Leupoldt and Dahme, 2005; Banzett et al., 2008).

Several medical conditions, such as chronic obstructive pulmonary disease (COPD), cardiac disease, cancer (Solano et al., 2006), and panic disorder (Smoller et al., 1996), are commonly associated with debilitating and frightening breathlessness. Breathlessness is well-described in motor neurone disease (Georges et al., 2016; Pattinson and Turner, 2016), and it is occasionally described in other neurological and neuromuscular conditions including Parkinson's disease (Bayulkem and Lopez, 2010), Multiple Sclerosis (Mutluay et al., 2005), and muscular dystrophy (Sivak et al., 1999). Often symptoms relate poorly to objective physiological measures of disease severity, such as spirometry (Jones, 2001; Herigstad et al., 2015). Functional brain imaging is beginning to reveal the brain mechanisms by which affective and emotional processes contribute to breathlessness (Herigstad et al., 2011, 2015; Hayen et al., 2013) and how peripheral respiratory input is integrated with higher consciousness (Ezra et al., 2015; Faull et al., 2015, 2016).

In healthy individuals, moderate to severe breathlessness is well-recognized as a normal sensation during intense exercise (Simon et al., 1989; Hamilton et al., 1996; Borg et al., 2010). These breathlessness sensations are usually considered “non-threatening” due to precise matching between expectation and the actual ventilatory response to heavy exercise (Laviolette and Laveneziana, 2014). However, as evidence continues to accumulate for the performance-limiting capacity of the ventilatory system in athletes (Powers et al., 1988; Johnson et al., 1992; Harms et al., 1997, 2000), it is possible that the affective components of breathlessness and the resultant anxiety when nearing maximal ventilation may become a key factor within the complex limitations of athletic performance in highly-trained individuals.

Ventilation during exercise is tightly controlled. A balance of neurally-modulated feed forward ventilatory commands and peripheral feedback stimulate increased ventilation that initially corresponds with exercise intensity (Kaufman and Forster, 1996; Waldrop et al., 1996). As exercise intensity increases, flux through the mitochondrial tricarboxylic-acid (TCA) cycle increases to match increased energy demands, increasing production of carbon dioxide ($\dot{\text{V}}{\text{CO}}_{2}$) (Wasserman et al., 1967). This linear relationship between exercise intensity and $\dot{\text{V}}{\text{CO}}_{2}$ continues until the anaerobic threshold, where $\dot{\text{V}}{\text{CO}}_{2}$ (and hence ventilation) increases disproportionately to the rate of oxygen consumed ($\dot{\text{V}}{\text{O}}_{2}$) and exercise intensity (Wasserman et al., 1973; Beaver et al., 1986). Deviation from this linear trend is currently understood to be due to the additional involvement of anaerobic metabolism, with increased respiration allowing buffering of lactic acid by ${\text{HCO}}_{3}^{-}$ ions that are excreted by the lungs as CO_2_ (Wasserman et al., 1973; Beaver et al., 1986).

During maximal exercise, the ability to “over-ventilate” beyond our metabolic needs maintains arterial-oxygen saturation (for exercising at sea level) in the majority of individuals, and thus ventilation itself was originally not considered a limiting factor to exercise performance (Bassett and Howley, 2000; McArdle et al., 2006). However, evidence now exists for both mechanical limitations of the lungs and respiratory muscles for inadequate alveolar ventilation in many elite athletes (Dempsey et al., 1984; Powers et al., 1988; Johnson et al., 1992), and a significant effect of respiratory muscle work on cardiac output to compromise peripheral muscle blood flow and exercise performance (Harms et al., 1997, 2000).

Alternatively, exertional breathlessness (although likely to be vastly more complex than in healthy individuals) can be induced even at very low intensities of exercise in people with respiratory disease, such as seen in COPD (Aldrich et al., 1982). Although maximal ventilation is impaired in COPD, the affective response to breathlessness at even low work rates has powerful limiting effects on performance. Particularly important is the breathlessness-related anxiety that is thought to drive behavioral changes and exacerbate symptoms, causing these individuals to cease exercising well before any significant ventilatory distress (Lansing et al., 2009; Herigstad et al., 2011, 2015; Janssens et al., 2011; Hayen et al., 2013). Therefore, if breathlessness-related anxiety can severely limit exercise performance in people with respiratory disease (Carrieri-Kohlman et al., 2001, 2010; Janssens et al., 2011), it seems plausible that highly-trained athletes who are capable of reaching maximal ventilory capacity during heavy exercise may also be affected by breathlessness-anxiety, albeit at much higher work rates, potentially contributing to performance limitations.

In this study, we examined whether endurance exercise-trained athletes differed in their intensity perception and anxiety of breathlessness during exercise compared with sedentary controls. Additionally, we employed a hypercapnic ventilatory response test to investigate whether the perceptual response to chemostimulated ventilation differed between the groups independent of exercise. Due to the importance of breathlessness-anxiety in determining exercise performance in people with clinical breathlessness, we hypothesized that athletes (who are known to operate at higher ventilatory capacities) may also present with greater breathlessness-anxiety during maximal exercise than sedentary controls. Understanding the relative contributions of both sensory and affective components of exercise-induced breathlessness may reveal a role of breathlessness-anxiety in athletic performance; an idea that has currently been overlooked in the current literature.

## Materials and methods

### Subjects

The Oxfordshire Clinical Research Ethics Committee approved the study and volunteers gave written, informed consent. In this study, we compared 20 endurance athletes and 20 age- and sex-matched sedentary subjects (20 males, 20 females; mean age ± SD, 26 ± 7 years; age-matched ± 2 years) in a cross-sectional design. The athlete group consisted of subjects who participated in endurance exercise training five or more times per week (cycling, rowing, distance running), while the sedentary group comprised subjects who were not involved in any organized exercise, and minimal commuting exercise. One athlete subject did not complete the maximal exercise test due to injury.

### Questionnaires

Due to the cross-sectional nature of this experiment, a thorough baseline comparison of general mood and anxiety between the two groups was necessitated, to assess any potential psychological influences on the ventilatory perceptions measured throughout the experiment. Self-report questionnaires were employed to assess any underlying differences in general trait anxiety, mood/depression or anxiety of bodily sensations. The questionnaires consisted of:

- Spielberger State-Trait Anxiety Inventory (STAI; Spielberger, 2010)
- Anxiety Sensitivity Index (ASI; Reiss et al., 1986)
- The Centre for Epidemiologic Studies Depression Scale (CES-D; Radloff, 1977)

Subjects were also asked to record how many hours of physical activity they typically completed per week, the intensity of the exercise (easy, moderate or intense) and what types of exercise they performed. They were asked to recall and record their last week of exercising activity.

### Visual analog scales for breathlessness and breathlessness-anxiety

Breathlessness and breathlessness-related anxiety were also measured during changes in ventilation associated with chemostimulated breathing and incremental exercise (outlined below). During both protocols, rating scores of breathlessness (“How breathless are you?”) were recorded at rest and after each stage, using a visual-analog scale (VAS) between “Not at all breathless” (0%) and “Most intense breathlessness imaginable” (100%). Subjects were also asked to rate how anxious they were about their breathing (“How anxious are you about your breathing?”), using a VAS between “Not at all anxious” (0%) and “Extremely anxious” (100%) immediately following the breathlessness scale at each stage.

### Spirometry

Spirometry was used to asses lung capacity and function, and was performed according to the Association for Respiratory Technology and Physiology standards (Levy et al., 2009). A full inspiration and expiration was used to measure forced vital capacity (FVC) of the lungs using three attempts, according to established guidelines (Levy et al., 2009). Maximal voluntary ventilation (MVV) was also tested to assess each subject's ability to ventilate of their own volition, where subjects were asked to ventilate maximally through the mouthpiece and turbine for 10 s. The best of two MVV measurements was recorded. Spirometry measurements were recorded using a mouth-piece (Hans Rudolf, Kansas City, MO, USA) and turbine connected to gas and flow analyser (Cortex Metalyser 3B, Cranlea Human Performance Ltd, Birmingham, UK), and subjects wore a nose clip for all spirometry tests. Metasoft studio software (Cortex, Versions 3.9.9 and 4.9.0, Cranlea Human Performance Ltd) was used to calculate all spirometry measurements.

### Breathlessness response to chemostimulated ventilation

**Figure 1:**
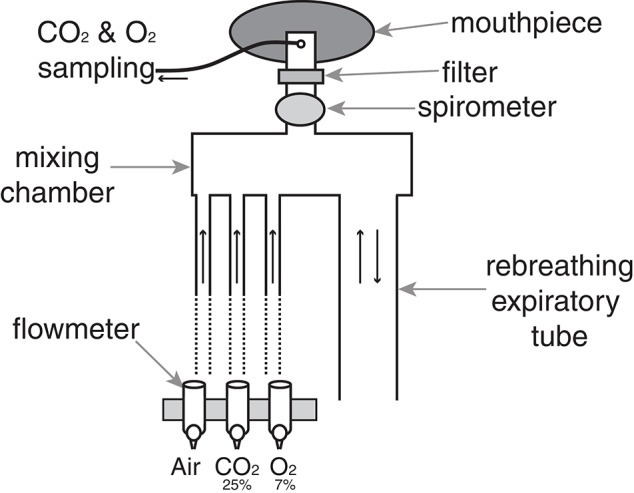
**Breathing system used to administer hypercapnic challenges**. At rest, humidified medical air is administered into the mixing chamber at a rate sufficient to avoid re-breathing of expired gases. A diving mouthpiece (Scubapro UK Ltd, Mitcham, UK) connects to a bacterial and viral filter (GVS, Lancashire, UK), and respiratory gases are sampled via polyethylene extension tubing (Vygon SA, Ecouen, France). A sampling line connects to a gas analyser that samples O_2_ and CO_2_ (Gas Analyser; ADInstruments Ltd, Oxford, United Kingdom), and end-tidal oxygen (P_ET_O_2_) and carbon dioxide (P_ET_O_2_) were monitored at all times using physiological monitoring software (Labchart 7; ADInstruments Ltd) on a computer monitor. During hypercapnia, the medical airflow was reduced and CO_2_ was delivered into the mixing chamber to adjust expired P_ET_CO_2_ to either 0.8 or 1.5% (randomized) above rest, and a hypoxic mixture was added to maintain P_ET_O_2_ at rest levels. A spirometer (ADInstruments Ltd) connected to a data acquisition device (Powerlab; ADInstruments Ltd, Oxford) simultaneously recorded ventilatory flow and volume within Labchart.

In this study we measured the ventilatory and subjective responses to a hypercapnic challenge, to better characterize any perceptual differences in ventilation between the groups. We maintained partial pressure of end-tidal oxygen (P_ET_O_2_) at resting levels by manually controlling the fraction of inspired oxygen in the gas mixture as ventilation increased. To administer the hypercapnic stimulus, a breathing system was designed to administer adjustable quantities of humidified medical air, carbon dioxide (CO_2_) and oxygen (O_2_), and gas sampling was displayed on a computer (using Labchart 7; ADInstruments Ltd, Oxford, United Kingdom) to monitor expired gases at all times (Figure 1). Expired gases were determined using a rapidly-responding gas analyser (Gas Analyser; ADInstruments Ltd, Oxford, United Kingdom), and ventilatory flow and volume were determined using a pneumotachograph (ADInstruments Ltd).

Subjects were positioned supine and attached to the breathing system. Eight minutes of resting breathing was initially recorded, with subjects rating their breathlessness and breathlessness-anxiety every 4 min using a VAS (explained above) presented on a computer screen and controlled by the participant via a button box. Resting values of P_ET_CO_2_ and P_ET_O_2_ were determined during this baseline period, before two levels of hypercapnia were administered. The hypercapnic periods involved a 3 min elevation in P_ET_CO_2_ of 0.8 (6.1 mm Hg) and 1.5% (11.2 mm Hg) above baseline (randomized order, separated by 4 min of breathing medical air) whilst P_ET_O_2_ was maintained at resting levels throughout the experiment (iso-oxia) through adjusting the concentration of oxygen in the inspired gas mixture, by carefully titrating appropriate amounts of a hypoxic gas (7% O_2_, balance nitrogen) into the inspirate according to individual subject's requirement. A final 4 min of breathing medical air followed the second hypercapnic stimulus to finish the test. Subjects were asked to rate their breathlessness and anxiety at the end of each block of hypercapnia and medical air. Hypercapnic ventilatory response was also calculated for each subject by regressing P_ET_CO_2_ values at rest and during the two levels of hypercapnia against the corresponding ventilations.

### Breathlessness response to exercise stimulated ventilation

To assess any possible changes in breathlessness perception as a result of exercise, subjects lastly performed an incremental sub-maximal to maximal exercise test with concurrent ventilatory and breathlessness measures. A relative sub-maximal point of reference of the “anaerobic threshold” was used for comparison between the two groups, as well as maximal exercise measures.

The incremental exercise test to exhaustion was completed on a stationary bicycle (Ergoline 500, Lindenstrasse, Germany), with subjects seated in a comfortable, upright position on standard handlebars. A facemask (Hans Rudolf) and turbine were connected to a gas and flow analyser (Cortex Metalyser 3B, Cranlea Human Performance Ltd; McLaughlin et al., 2001) to measure expired gases and ventilatory flow on a breath-by-breath basis, with a pneumotach sampling rate exceeding 500 Hz (output averaged over 15 s; Metasoft studio software, Cortex, Versions 3.9.9 and 4.9.0, Cranlea Human Performance Ltd). Exercise began between 50 and 150 W (depending on predicted $\dot{\text{V}}{\text{O}}_{2\text{peak}}$) at a self-selected cadence (target of 90 rpm), and 3 min stages of 50 W increments were completed until exhaustion. These stages were chosen to allow steady-state assessment of breathlessness at each exercise intensity. Breathlessness and breathlessness-anxiety were rated on a 0–100% VAS scale (as described above) at the beginning of the exercise test, in the last 30 s of each stage and at exhaustion. Anaerobic threshold was determined by visual inspection using the V-slope method (Wasserman et al., 1973; Beaver et al., 1986).

### Statistics

All data are presented as means ± standard deviation. Statistical analysis was performed using SPSS (Version 21, SPSS, IBM, Armonk, New York, USA), and statistical significance was established *a-priori* at *p* < 0.05 using a Student's independent (unpaired) samples *t*-test.

## Results

### Baseline physiology and psychology

All physiological and psychological baseline measures are presented in Table 1. Athletes were significantly taller, with larger lung vital capacities and MVV. No differences were observed between the groups for any baseline psychological measures.

**Table 1:** **Mean ± SD baseline group physiology measures and questionnaires**.

	**Athlete**	**Sedentary**	
Females/Males	10/10	10/10	
Training volume (hours/week)	11.5 (0.7)	0	
Age (years)	25.8 (7.5)	25.7 (7.4)	*p* = 0.95
Height (m)	1.8 (0.9)	1.7 (0.1)	*p* = 0.01^*^
Weight (kg)	75.2 (10.1)	68.7 (13.6)	*p* = 0.09
BMI (kg/m2)	23.1 (2.8)	23.3 (3.5)	*p* = 0.87
FVC (L)	5.7 (0.9)	4.2 (1.2)	*p* < 0.001^*^
FVC (% predicted)	109.5 (9.4)	91.0 (19.5)	*p* < 0.001^*^
FEV1/FVC (%)	78.2 (7.0)	81.3 (4.6)	*p* = 0.10
MVV (L/min)	150.9 (42.8)	113.0 (39.5)	*p* = 0.01^*^
Exercise (volume x intensity)	20.3 (6.0)	1.8 (1.9)	*p* = 0.001^*^
Trait anxiety	29.6 (5.9)	30.8 (6.8)	*p* = 0.54
Pre-exercise state anxiety	27.8 (6.5)	25.6 (5.4)	*p* = 0.25
Anxiety sensitivity index	13.5 (6.1)	16.1 (7.7)	*p* = 0.24
Depression	6.4 (4.2)	7.6 (4.7)	*p* = 0.40

*BMI, body mass index; FVC, forced vital capacity; FEV1/FVC, forced expiratory volume in 1s as a fraction of forced vital capacity; MVV, maximal voluntary ventilation. Exercise exposure (“Exercise”) was measured as the number of hours per week x intensity of exercise (1–3: easy, moderate, intense)*.

* *Significantly different (p < 0.05) between groups*.

### Breathlessness response to exercise stimulated ventilation

**Figure 2:**
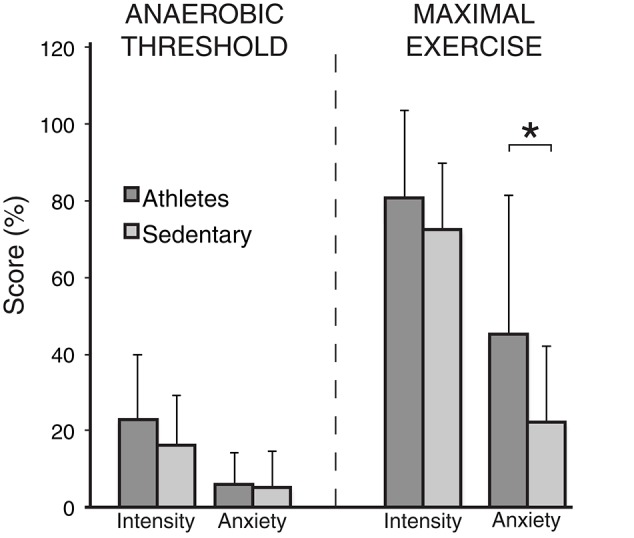
**Breathlessness intensity and anxiety scores (on a scale of 0−100%) at anaerobic threshold and maximal exercise for both athletes and sedentary subjects**. Bars represent group mean, and error bars standard deviation. “Intensity” represents intensity of breathlessness, and “Anxiety” represents breathlessness-anxiety. ^*^Significantly different between the groups (*p* < 0.05).

**Figure 3:**
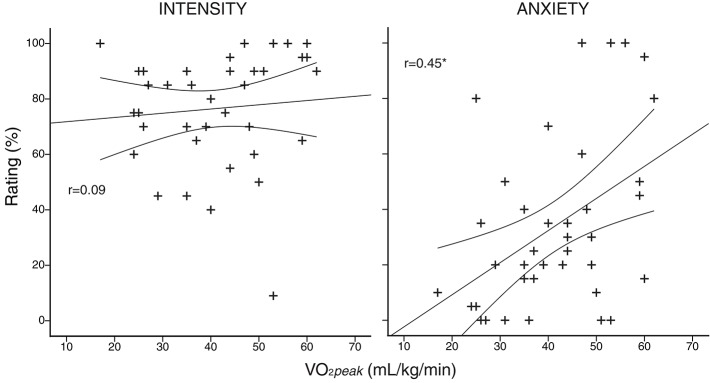
**Subject-specific anxiety and intensity scores plotted against peak volume of oxygen consumption (V°O_*2peak*_), measured during an incremental exercise test to exhaustion on a stationary bicycle**. Intensity of breathlessness is plotted in the left column, and anxiety of breathlessness in the right column. ^*^r correlation coefficient significant (*p* < 0.05). Curved lines represent 95% confidence intervals of the regression.

A summary of the physiological and psychological variables measured during incremental exercise is presented in Table 2. As expected, both work rate and VO_2_ were greater in athletes at both anaerobic threshold and maximal exercise, and the anaerobic threshold of athletes was at a greater percentage of their maximum. At maximal exercise, the absolute and relative increase in ventilation from baseline was greater in athletes, and peak volume of oxygen consumption (VO_2peak_) was larger in the athlete group. At maximal exercise, athletes ventilated at a greater proportion of their MVV than their sedentary counterparts. For the psychological variables, while there was no difference in breathlessness intensity values at maximal exercise between the groups, athletes rated significantly higher in breathlessness-anxiety than sedentary subjects (Figure 2). Further investigation revealed that breathlessness-anxiety at maximal exercise positively correlated with measured VO_2peak_, while breathlessness intensity did not (Figure 3). A *post-hoc* power calculation was performed to provide an initial estimation of the effect size in this pilot study, and produced a *d*_*cohen*_ = 0.79 for differences in breathlessness-anxiety at maximal exercise, falling just below the conventional “large” effect size threshold (Cohen, 1992).

**Table 2:** **Physiological and psychological variables during rest and incremental exercise test to exhaustion on a cycle ergometer (mean ± SD)**.

**Anaerobic threshold**	**Athlete**	**Sedentary**	
Work rate (W)	219.7 (46.8)	102.6 (24.9)	*p* < 0.001^*^
Work rate (% of max)	67.6 (7.7)	59.7 (12.8)	*p* = 0.03^*^
$\dot{\text{V}}{\text{O}}_{2}$ (mL/kg/min)	38.4 (7.7)	20.3 (4.5)	*p* < 0.001^*^
${\dot{\text{V}}}_{\text{E}}$ (L/min)	76.7 (17.0)	38.9 (10.1)	*p* < 0.001^*^
${\dot{\text{V}}}_{\text{E}}$ (% of max)	53.4 (10.0)	52.6 (13.3)	*p* = 0.84
Breathlessness intensity (%)	22.9 (17.1)	16.1 (13.2)	*p* = 0.17
Breathlessness-anxiety (%)	5.9 (8.2)	5.2 (9.6)	*p* = 0.78
**Maximal exercise**	**Athlete**	**Sedentary**	
Work rate (W)^*^	325.0 (59.5)	173.8 (45.5)	*p* < 0.001^*^
$\dot{\text{V}}{\text{O}}_{2\text{peak}}$ (mL/kg/min)^*^	50.8 (7.3)	31.6 (7.4)	*p* < 0.001^*^
${\dot{\text{V}}}_{\text{E}}$ (L/min)	146.4 (37.0)	77.8 (27.0)	*p* < 0.001^*^
${\dot{\text{V}}}_{\text{E}}$ (% above baseline)^*^	1051.7 (276.0)	665.7 (298.2)	*p* < 0.001^*^
${\dot{\text{V}}}_{\text{E}}$ (% MVV)^*^	101.6 (27.2)	73.7 (30.1)	*p* = 0.03^*^
Breathlessness intensity (%)	80.7 (22.7)	72.5 (16.4)	*p* = 0.21
Breathlessness-anxiety (%)	45.3 (36.3)	22.3 (20.6)	*p* = 0.02^*^

*$\dot{\text{V}}{\text{O}}_{2}$, volume of oxygen consumed; ${\dot{\text{V}}}_{E}$, ventilation; MVV, maximal voluntary ventilation*.

* *Significantly different (p < 0.05) between groups*.

### Breathlessness response to chemostimulated ventilation

Mean group values for end-tidal gases, ventilation and subjective ratings of breathlessness during rest and the two steps of the hypercapnic challenge are presented in Table 3. Ventilation is presented as both an absolute value and percentage change from baseline (Table 1). No mean differences were observed between groups for subjective ratings of either intensity and anxiety of breathlessness at both levels of hypercapnia, and P_*ET*_O_2_ remained stable across the entire experiment in both groups.

**Table 3:** **Physiological and psychological variables during rest and two levels of a hypercapnic hyperventilatory challenge (mild hypercapnia: aim of 0.8% (6.1 mm Hg); and moderate hypercapnia: aim of 1.5% (11.2 mm Hg) increase in P_*ET*_CO_2_; mean ± SD)**.

**Rest**	**Athlete**	**Sedentary**	
P_ET_CO_2_ (mmHg)	38.9 (5.1)	40.1 (3.5)	*p* = 0.39
P_ET_O_2_ (mmHg)	116.3 (7.7)	115.7 (6.5)	*p* = 0.79
${\dot{\text{V}}}_{\text{E}}$ (l/min)	13.0 (3.6)	10.5 (2.5)	*p* = 0.01^*^
Intensity rating (%)	3.1 (4.5)	4.6 (3.8)	*p* = 0.25
Anxiety rating (%)	3.1 (4.1)	5.1 (5.1)	*p* = 0.17
**Mild hypercapnia**	**Athlete**	**Sedentary**	
P_ET_CO_2_ (mmHg)	46.0 (4.7)	46.5 (3.0)	*p* = 0.67
P_ET_O_2_ (mmHg)	114.7 (5.6)	115.8 (4.6)	*p* = 0.49
${\dot{\text{V}}}_{\text{E}}$ (l/min)	23.3 (10.2)	20.6 (4.8)	*p* = 0.30
Change in ${\dot{\text{V}}}_{\text{E}}$ (%)	87.2 (84.7)	104.3 (58.5)	*p* = 0.46
Intensity rating (%)	26.0 (20.2)	21.9 (15.5)	*p* = 0.48
Anxiety rating (%)	18.9 (17.9)	17.2 (13.9)	*p* = 0.74
**Moderate hypercapnia**	**Athlete**	**Sedentary**	
P_ET_CO_2_ (mmHg)	50.6 (4.8)	51.2 (3.3)	*p* = 0.63
P_ET_O_2_ (mmHg)	115.9 (4.6)	117.6 (6.0)	*p* = 0.32
${\dot{\text{V}}}_{\text{E}}$ (l/min)	34.0 (14.6)	31.1 (11.4)	*p* = 0.49
Change in ${\dot{\text{V}}}_{\text{E}}$ (%)	174.4 (115.4)	205.6 (121.4)	*p* = 0.41
Intensity rating (%)	41.9 (24.4)	47.8 (12.4)	*p* = 0.34
Anxiety rating (%)	36.5 (25.8)	32.3 (16.2)	*p* = 0.54

*The order of hypercapnia levels was randomized between subjects, and results are presented as mean ± SD.V°O_2_, volume of oxygen consumed; ${\dot{\text{V}}}_{\text{E}}$, ventilation; MVV, maximal voluntary ventilation*.

* *Significantly different (p < 0.05) between groups*.

Hypercapnic ventilatory response (HCVR) was calculated using a linear regression between ventilation and P_ET_CO_2_, and no difference was found between the groups (mean ± SD): athletes 13.7 (9.6) vs. sedentary 14.0 (7.4) (l/min/% change in CO_2_). For visual representation of individual subject HCVR see Supplementary Figure 1.

**Figure 4:**
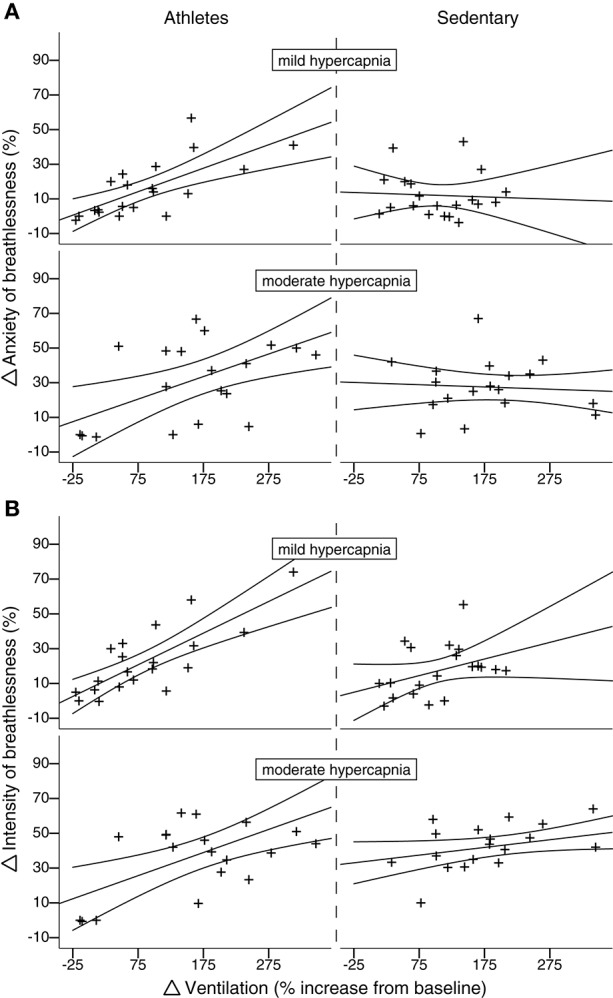
**Subject-specific change in breathlessness-anxiety (A) and intensity (B) scores plotted against percentage change in ventilation from baseline induced by both mild and moderate hypercapnia (mild hypercapnia: aim of 0.8%; and moderate hypercapnia: aim of 1.5% increase in P_*ET*_*O*_2_)**. Athletes are plotted in the left column, and sedentary subjects in the right column. For moderate (but not mild) hypercapnia, athletes display a linear correlation between change in ventilation and change in intensity score that significantly differs from the null relationship seen in sedentary subjects. Ninety five percent confidence intervals are shown.

As the hypercapnic ventilatory response did not differ between the two groups, and there was no relationship between HCVR and either breathlessness intensity or anxiety (*P* > 0.05), we were then able to investigate the relationship between isolated changes in ventilation and the corresponding anxiety/intensity scores. During both mild and moderate hypercapnia, the athlete group showed a positive linear correlation for both intensity and anxiety with ventilation (mild hypercapnia: intensity vs. ventilation: slope = 5.41; *r* = 0.79; *p* < 0.05; anxiety vs. ventilation: slope = 4.21; *r* = 0.70; *p* < 0.05; moderate hypercapnia: intensity vs. ventilation: slope = 3.52; *r* = 0.64; *p* < 0.05; anxiety vs. ventilation: slope = 3.10; *r* = 0.59; *p* < 0.05), while the sedentary group did not (mild hypercapnia: intensity vs. ventilation: slope = 1.75; *r* = 0.38; *p* > 0.05; anxiety vs. ventilation: slope = −0.25; *r* = 0.06; *p* > 0.05; moderate hypercapnia: intensity vs. ventilation: slope = 2.00; *r* = 0.43; *p* > 0.05; anxiety vs. ventilation: slope = −0.45; *r* = 0.11; *p* > 0.05). These linear correlations with ventilation observed in athletes were significantly greater than sedentary subjects for both intensity and anxiety in moderate hypercapnia, and for anxiety only in mild hypercapnia (Figure 4; Supplementary Table 1).

## Discussion

### Main findings

In this pilot study, we found that athletes reported increased anxiety of breathlessness compared to sedentary subjects at maximal exercise, while breathlessness intensity was rated at a similar level between the groups. Correspondingly, athletes were also found to be operating at a greater percentage of their MVV at maximal exercise. During a hypercapnic ventilatory response test, we found that athletes' breathlessness scores more accurately matched ventilation.

### Breathlessness and exercise

By separately assessing breathlessness intensity and anxiety, we can begin to disentangle the effects of sensory and affective components of breathlessness during exercise. While previous studies examining exercising breathlessness have used a Borg or modified Borg scales for breathlessness (El-Manshawi et al., 1986; Hamilton et al., 1996; Borg et al., 2010), this scale disregards the affective component of breathlessness, which is known to be important in clinical populations (Lansing et al., 2009; Herigstad et al., 2011, 2015, 2016; Janssens et al., 2011; Hayen et al., 2013). Interestingly, the increase in breathlessness-anxiety was not apparent at anaerobic threshold in athletes, indicating that this is not a general breathlessness hypersensitivity, but rather limited to maximal efforts. Correspondingly, the ventilation reached during maximal exercise in athletes was a significantly greater proportion of measured MVV than sedentary subjects. This may be due to improved muscular metabolism and exercising capacity to place greater demands on the cardiorespiratory system, as this was also paired with a significantly greater increase in ventilation in the athlete group. Therefore, reaching a higher proportion of MVV may result in conflicting feedback between a greater drive to breathe and the strain of ventilatory muscle work (Schwartzstein et al., 1989; Waldrop et al., 1996; Kaufman and Forster, 1996), contributing to an increase in breathlessness-anxiety (Altose, 1985).

#### Performance implications

While it is now known that mechanical limitations of the lungs and respiratory muscles can be reached in elite athletes during exercise (Dempsey et al., 1984; Powers et al., 1988; Johnson et al., 1992), and that respiratory muscle work can indirectly impede performance (Harms et al., 1997, 2000), perceptions of leg fatigue have previously been considered more important than breathlessness during maximal exercise (Borg et al., 2010). However, the effect of the emotional component of breathlessness on performance is not often considered. While the breathlessness-anxiety scores reported in this study were lower than those of breathlessness intensity, breathlessness-anxiety scores were found to correlate with V°O_2peak_ across all subjects (Figure 3). Therefore, it is possible that breathlessness-anxiety may become a greater influence on performance in athletes with greater aerobic capacities than those tested in the current study.

Alternatively, the broad range of breathlessness-anxiety scores reported at maximal exercise may represent individuals who are more or less susceptible to affective breathlessness-anxiety, and a subset of athletes for whom breathing perception may play an important role in performance. It is possible that athletes who are genetically endowed with lower respiratory muscle endurance, or a higher sensitivity to ventilatory signals are also at risk of breathlessness-anxiety limiting their performance. Further research into the brain processing of sensory and affective components of breathlessness within the sensorimotor and limbic systems of the brain (Faull et al., 2015, 2016), together with ventilatory physiology is needed to better understand the underlying mechanisms of exercising breathlessness intensity and anxiety. It is also possible that psychophysiological interventions focused on reducing the impact of breathlessness-anxiety on performance may be advantageous to those susceptible athletes (reviewed by Gardner and Moore, 2004). Therefore, while debate thrives as to whether peripheral or central factors limit exercise performance (Bassett and Howley, 2000), we have revealed a potential place for breathlessness-anxiety in highly-trained, susceptible individuals. While we are not suggesting that anxiety of breathlessness is the sole limiting factor to performance, it may be an indicator of nearing ventilatory capacity and a possible area for targeted improvement in predisposed athletes.

### Breathlessness and chemostimulated ventilation

Although we did not find group differences in ventilatory response or breathlessness scores during hypercapnia stimulated ventilation, further investigation revealed that in the athlete group breathlessness scores positively correlated with the change in ventilation, which was not observed in the sedentary subject group. This relationship between the physiological change in ventilation and subjective breathlessness implies a more finely-tuned perception of breathing changes in athletes. The differences in interoception may result from repeated exposure to elevated ventilation and breathlessness during endurance exercise training, and could potentially contribute to the elevated anxiety at maximal exercise demonstrated by athletes, as athletes may be more acutely aware of their ventilatory work approaching maximal capacity.

#### Performance implications for athletes

The closer matching of ventilatory responses to breathlessness in athletes found in this study may reflect better monitoring of exercise intensity and pacing during sporting performance. Fatigue of ventilatory muscles has been suggested as an important factor in endurance exercise, due to the competition for blood and metabolites with the working peripheral muscles (Johnson et al., 1996; Harms et al., 2000; Romer and Polkey, 2008), and thus appropriate pacing may reduce this impact on performance. Further studies on ventilatory interoception (e.g., adapted from those employed in cardiovascular interoception; Garfinkel et al., 2015) in athletes may help us to better understand these psychological contributions to performance.

#### Clinical implications

The results of this study may also help us to speculate more deeply on the mechanisms of breathlessness in clinical populations. It is well-recognized that people who suffer breathlessness may have altered perception of their breathing (Giardino et al., 2010). Due to the learned associations between breathlessness and disease, maladaptive learning may then contribute to increased breathlessness-anxiety (Ley, 1999). It is thought that through repeated exposure to breathlessness, those with respiratory disease begin to fear and avoid activities that may induce this sensation, resulting in further deterioration of the ventilatory muscles in the chest and further reduction in MVV (Herigstad et al., 2011; Hayen et al., 2013). In an elegant review by Smoller et al. (1996), the intricacy of the relationship between physiological and subjective sensations of ventilation in panic anxiety, hyperventilation and breathlessness is outlined. Smoller et al. (1996) explains that the presence of chronic breathlessness can be considered a risk factor for anxiety and panic attacks, while panic disorder patients also report debilitating sensations of breathlessness that exacerbate their anxiety (Smoller et al., 1996). It is therefore possible that an inappropriately tuned perception toward ventilation (as seen in athletes) due to repeated breathlessness exposure is a common contributing factor to these classes of disease.

### Study design

Due to the underlying physiological differences between athletes and sedentary subjects investigated in this study, considerations were taken to account for fundamental differences between the groups when attempting to make breathlessness comparisons. The exercising timepoints used for comparisons were maximal exercise and anaerobic threshold, with these comparable relative exercise intensities providing a more consistent platform from which to examine perceptual differences in breathlessness.

This pilot study is one of the first to consider perceptions of both breathlessness intensity and breathlessness-anxiety during exercise and hyrpercapnia-induced changes in ventilation in healthy individuals. The sample size of 40 subjects limits the generalisability of these findings, and further investigations involving greater numbers of subjects over a wide variety of training statuses would be highly beneficial for exploring the intricacies of how breathlessness perceptions may influence exercising performance.

## Conclusions

This study showed that athletes report increased breathlessness-anxiety at maximal exercise compared to sedentary subjects, while intensity of breathlessness remains similar between the groups. We postulate that this may be related to athletes reaching a greater proportion of their maximal ventilatory capacity. While the intensity of perceived breathlessness has previously not been considered a contributor to maximal exercise, this affective dimension of breathlessness-anxiety may represent both a performance limitation and a clinically important homeostatic signaling system for exercising breathlessness in susceptible, highly-trained individuals. Furthermore, we propose that the significantly different, more linear relationship between changes in ventilation and subjective breathlessness scores in athletes compared to sedentary subjects represents differences in ventilatory perception, which may also contribute to the increased breathlessness-anxiety reported at maximal exercise.

## Author contributions

OF and PC were involved in the experimental design, data collection and manuscript preparation. KP is the senior author and was involved in study design and manuscript preparation.

### Conflict of interest statement

The authors declare that the research was conducted in the absence of any commercial or financial relationships that could be construed as a potential conflict of interest.
