# Supplementary material for: Psychophysical Differences in Ventilatory Awareness and Breathlessness between Athletes and Sedentary Individuals
Source: Front Physiol. 2016 Jun 16;7:231. doi: 10.3389/fphys.2016.00231 (PMC4910254; doi:10.3389/fphys.2016.00231)
Supplement: Supplementary file 1 [file DataSheet1.docx]

***Supplementary Material***

**Psychophysical differences in ventilatory awareness and breathlessness between athletes and sedentary individuals**

Olivia K. Faull*^1,2,3^, Pete Cox^3^, Kyle T. S. Pattinson^1,2^

^1^ FMRIB Centre, ^2^ Nuffield Division of Anesthetics, Nuffield Department of Clinical Neurosciences, University of Oxford, Oxford, UK and ^3^ Department of Physiology, Anatomy and Genetics, University of Oxford, Oxford, UK

**Corresponding author:**

Dr Olivia Faull

Nuffield Department of Clinical Neurosciences

University of Oxford

Oxford, UK

Email: [olivia.faull@ndcn.ox.ac.uk](mailto:olivia.faull@ndcn.ox.ac.uk)

Phone: +44 (0)1865 34544

Fax: +44 (0)1865 23079

*Supplementary Figure 1: Demonstration of the regression slopes between ventilation and end-tidal CO*2 *in each of 20 amateur endurance (A) athletes and 20 healthy sedentary subjects (B).*

*Supplementary Table 1: Regression (slope and regression coefficient) of changes in ventilation against subjective scores of intensity and anxiety of breathlessness, during mild and moderate hypercapnia (mild hypercapnia: aim of 0.8%; and moderate hypercapnia: aim of 1.5% increase in end-tidal CO*2*). *Significant regression coefficient; and **significant regression coefficient that differs from sedentary group (p*<*0.05).*

| Mild hypercapnia | Athlete | Sedentary |
| --- | --- | --- |
| Intensity vs ventilation | 5.41 (0.79)* | 1.75 (0.38) |
| (slope (regression coefficient; R)) |  |  |
| Anxiety vs ventilation | 4.21 (0.70)** | -0.25 (0.06) |
| (slope (regression coefficient; R)) |  |  |
| Moderate hypercapnia | Athlete | Sedentary |
| Intensity vs ventilation | 3.52 (0.64)** | 2.00 (0.43) |
| (slope (regression coefficient; R)) |  |  |
| Anxiety vs ventilation | 3.10 (0.59)** | -0.45 (0.11) |
| (slope (regression coefficient; R)) |  |  |
